# Supplementary material for: Vulcan: Improved long-read mapping and structural variant calling via dual-mode alignment
Source: Gigascience. 2021 Sep 24;10(9):giab063. doi: 10.1093/gigascience/giab063 (PMC8463296; doi:10.1093/gigascience/giab063)
Supplement: giab063_Supplemental_File [file giab063_supplemental_file.pdf]

Supplementary Info

Supplementary Table 1: Programs, program versions, and parameters used in this study.

Program	Version	Usage	Parameters
Vulcan	1.0.2	read mapping	vulcan [] -t threads
minimap2	2.17-r941	simulated ONT read mapping	minimap2 --MD -x map-ont -a
minimap2	2.17-r941	Human ONT read mapping	minimap2 --MD -x map-ont -a -z 600,200
minimap2	2.17-r941	Human PacBio HiFi read mapping	minimap2 --MD -a -k 19 -O 5,56 -E 4,1 -B 5 -z 400,50 -r 2k –eqx –secondary=no
minimap2	2.17-r941	Human Pacbio CLR read mapping	minimap2 --MD -x map-pb -a –eqx -L -O 5,56 -E 4,1 -B 5 –secondary=no -z 400,50 -r 2k -Y
NGMLR	0.2.7	read mapping	NGMLR -x [ont | pacbio] –bam-fix
Sniffles	1.0.12	SV calling	sniffles -s 2
bcftools	1.7	VCF filtering	bcftools view -i (INFO/SVLEN>=50|INFO/SVLEN<=-50|INFO/SVLEN=0|INFO/SVLEN=1)&(INFO/RE={read_num})
Truvari	v2.0.0-dev	SV benchmarking	truvari bench -b {GIAB_vcf} -c {sorted_gzipped_vcf} -f {reference_genome} –passonly–giabreport –pctsim=0 –multimatch –includebed {GIAB_bed} -o {benchmarking_output}
SURVIVOR	1.0.7	SV simulation	SURVIVOR simSV {reference_genome} {parameter_file} 0.01 0 {output_prefix}

Supplementary Table 2: Accuracy, Precision and F1 score of simulated data.

Nanopore Simulated 10X	Recall	Precision	F1
minimap2	81%	62%	70.24%
NGMLR	92%	62%	74.24%
Vulcan 80%	**95%**	**74%**	**83.13%**

Supplementary Figure 1: Wall time benchmarking of Vulcan, NGMLR and minimap2 on Nanopore 10X datasets

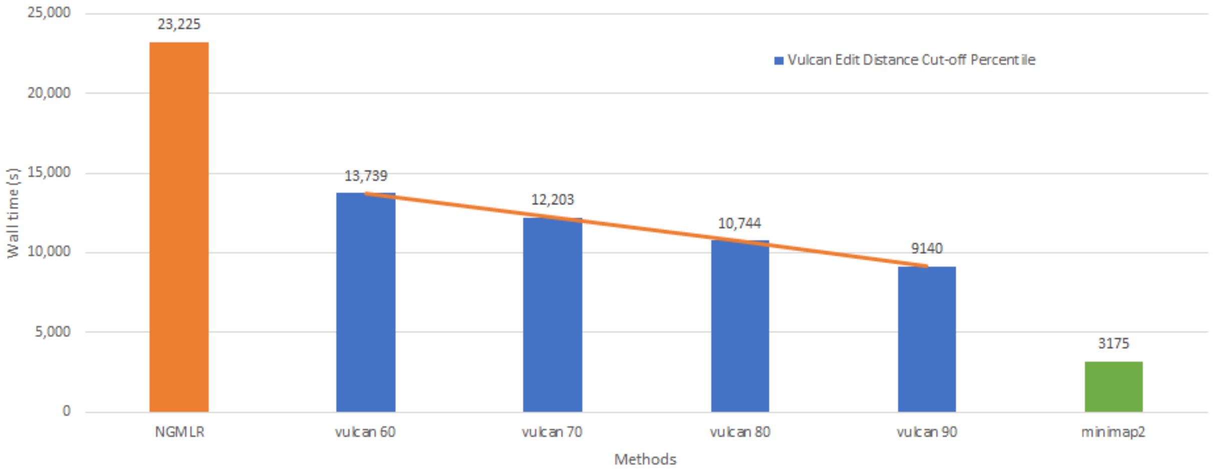

A wall time benchmarking has been performed to compare the performance of three different methods. From the chart we can infer that Vulcan takes less than 2/5 time than NGMLR. The experiment was performed on a Nanopore 10X subsmaple real dataset from the GIAB project.

Supplementary Figure 2: CPU time benchmarking of Vulcan, NGMLR and minimap2 on PacBio 20X datasets

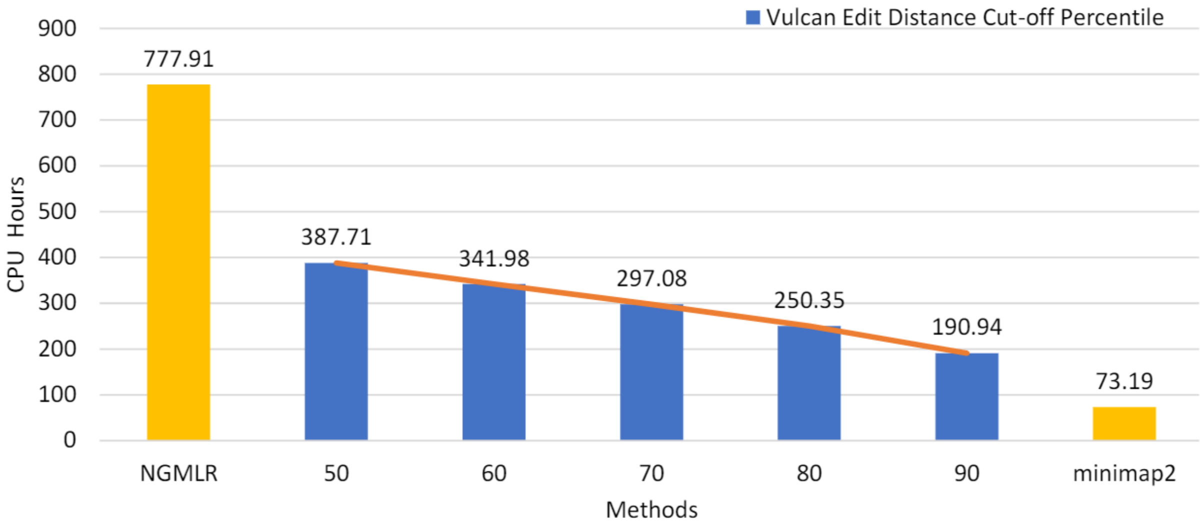

Vulcan achieves an approximately linear acceleration with the increase of the cut-off percentile. With a 90% percentile cut-off, Vulcan only takes about 1/4 of NGMLR's runtime. The experiment was performed on a PacBio CLR 20X subsmapled real dataset from the GIAB project.
