# Supplementary material for: Vulcan: Improved long-read mapping and structural variant calling via dual-mode alignment
Source: Gigascience. 2021 Sep 24;10(9):giab063. doi: 10.1093/gigascience/giab063 (PMC8463296; doi:10.1093/gigascience/giab063)
Supplement: giab063_Response_to_Reviewer_Comments_Original_Submission [file giab063_response_to_reviewer_comments_original_submission.pdf]

Author's Response To Reviewer Comments

Close

Reviewer responses attached as supplementary file titled "Vulcan_reviewer_responses.pdf"

Close
