# Supplementary material for: Vulcan: Improved long-read mapping and structural variant calling via dual-mode alignment
Source: Gigascience. 2021 Sep 24;10(9):giab063. doi: 10.1093/gigascience/giab063 (PMC8463296; doi:10.1093/gigascience/giab063)
Supplement: giab063_Response_to_Reviewer_Comments_Revision_1 [file giab063_response_to_reviewer_comments_revision_1.pdf]

Author's Response To Reviewer Comments

Close

[Editorial comments]

- address the (very minor) additional comments of the reviewers (see below).

R1: additional comments addressed in revised version (changes indicated in red).

- register any new software tool in the bio.tools and SciCrunch.org databases to receive RRID (Research Resource Identification Initiative ID) and biotoolsID identifiers, and include these in your manuscript (in the "code availability" section). This will facilitate tracking, reproducibility and re-use of your tool.

R2: Software registered as:
1. SciCrunch.org database with id SCR_021657
2. Bio.tools with id biotools:vulcan_mapper

- move URLs and ftp sites for data sources, software, etc. to the bibliography, and cite them by number from the text. Please refer to our citation guidelines in our "instructions for authors".

R3: URLS now cited

- our curators will contact you shortly to prepare the supporting data (an archival snapshot of the code) for release via our database GigaDB. Please include this GigaDB data set in the bibliography and cite it by number from the "availability" section.

R4: GigaDB linked

- please include ORCIDs on the title page, where available.

R5: ORCIDs added

[Reviewer 1]

1. Page 4: "CPU time was measured in CPU time for all programs" -> perhaps it meant to be "The time was measured in terms of CPU time for all programs"?

Fixed

2. Fig 3 text is too small to see on the print. Please enlarge them to a size similar to Fig 2.

Fig3 text enlarged

3. Supplementary: -MD flag is still missing I think.

Added –MD tag of minimap2 in supplementary

[Reviewer 2]

1. Correct spit reads -> split reads in the newly added sentence: "The soft clipping also takes place at spit reads that are indicative for SV and thus often form at breakpoints of SV.".

Fixed

2. The sub-heading, "Vulcan Accelerates Long-read Mapping for SV Calling", uses "SV Calling". I'd

suggest making this consistent with the rest of the subheadings.

Subheading modified and made consistent

Close
