# Supplementary material for: Vulcan: Improved long-read mapping and structural variant calling via dual-mode alignment
Source: Gigascience. 2021 Sep 24;10(9):giab063. doi: 10.1093/gigascience/giab063 (PMC8463296; doi:10.1093/gigascience/giab063)
Supplement: giab063_Reviewer_1_Report_Original_Submission — Hasindu Gamaarachchi -- 6/28/2021 Reviewed [file giab063_reviewer_1_report_original_submission.pdf]

Reviewer Report

Title: Vulcan: Improved long-read mapping and structural variant calling via dual-mode alignment

Version: Original Submission **Date:** 6/28/2021

Reviewer name: Hasindu Gamaarachchi

Reviewer Comments to Author:

This paper aims to improve the accuracy and the computational cost of the increasingly important yet immature area of structural variant calling using long reads. The proposed method has considerably improved the F1 measure for structural variant calling on a real nanopore dataset by 2-5%. However, the performance improvement has been marginal and sometimes even slightly detrimental for PacBio datasets.

The paper is well written, well-structured, and easy to follow. Vulcan - the software provided as open-source - was fairly easy to install. Although my first attempt to install the software failed due to an outdated conda version, the installation was smooth after I updated Conda to the latest. I then successfully ran Vulcan on a NA12878 PromethION dataset (30X coverage) on a server with 32 cores. I could not find a truth set for structural variants on NA12878 and thus could not verify the accuracy claims myself, but I trust the authors on this.

The major concern is about the presented runtime information.

1. The authors have provided the runtime information only for the PacBio data, despite their tool being mostly useful for nanopore in terms of accuracy. As the accuracy benefits are mainly for nanopore, runtime information for nanopore also should be included. Characteristics of nanopore data are different to Pacbio and the runtime performance tends to be different as it has been for accuracy. So, the authors are advised to perform runtime benchmarks on the nanopore datasets as well and include them in the paper.
2. The authors have used the CPU time as a metric to measure the runtime and the speedup which can be deceiving. Instead, wall-clock time is a better metric to measure the runtime and speedup, especially in multi-threaded programmes. Time spent on I/O, locks such as semaphores, mutexes and conditional variables and time spent by threads in the sleep state are not included in the CPU time. From a user's perspective, the wall-clock time would be of greater importance than the CPU time. On the other hand, CPU time is more indicative of how "processor intensive" a programme is and together with wall-clock time can be used to estimate the CPU utilisation percentage. The authors are requested to measure the wall-clock times and include them instead or alongside the CPU times.

The above two concerns are aggravated when observing the runtimes that I got for nanopore data which are a bit different to the values indicated on the paper (assuming I ran the commands correctly):

```
vulcan -ont -t 32 -r ref.fa -i reads.fastq -w vulcan_tmp -o ./output-vulcan/vulcan
    User time (seconds): 1707100.12
    System time (seconds): 18088.83
    Elapsed (wall clock) time (h:mm:ss or m:ss): 17:47:00
```

ngmlr -x ont --skip-write -t 32 -r ref.fa -q reads.fastq -o reads.sam
User time (seconds): 5403019.18
System time (seconds): 25675.86
Elapsed (wall clock) time (h:mm:ss or m:ss): 47:31:41
minimap2 -x map-ont -a --MD -t32 ref.fa reads.fastq
User time (seconds): 202131.44
System time (seconds): 6901.30
Elapsed (wall clock) time (h:mm:ss or m:ss): 2:00:20

The actual speedup (wall-clock time-based) of Vulcan over NGLMR is around 2.7 opposed to 4X. And, Vulcan takes around 8.2 times the runtime of minimap2 as opposed to 2.5X. If the CPU time ratios are used as authors did for the speedup (5403019.18+25675.86)/(1707100.12+18088.83) = 3.15X and (1707100.12+18088.83)/(202131.44+6901.30) = 8.23, respectively.

Following are some other issues and some doubts I had:

Page 3
- Why was 10X coverage used for simulated data? How would it behave for 20X and 30X? Why is 10X accuracy values for simulations look a bit different to real 10X data?
- Analyses: Why was hg19 which is an older version of the reference used. The truth set is also not in hg38 but I am wondering if this can be lifted over?

Page 4-5
It would be good to state that "normalized edit distance" is defined under methods. The first time I came across this "normalized edit distance", I wondered if this is an z-score normalisation and then later found the definition under methods.

Page 6
- 4X speedup over NGLMR: speed up is usually for wall-clock time not for CPU time as mentioned above
- the term runtime usually means the wall-clock time not the CPU time
- only the RAM usage for Vulcan with PacBio 20X is given. How is the RAM usage for Minimap2 alone and HGMLR alone? Also, how is the RAM usage for nanopore data, which is more important as Vulcan mainly benefits the accuracy on nanopore?
- execution also refers to wall-clock time, not the CPU time.

Page 7
- "We will next describe SV performance for various Nanopore coverages (20x, 30x, 50X), PacBio CLR (20x), and PacBio HiFi (10X) datasets": these values are inconsistent with what is in the results (for instance Table 1)
- "Similar to the simulated data, we achieve the best F1 scores using Vulcan together with Sniffles." : What are the numbers for F1 scores. If I did not miss, the exact F1 scores for simulated data are not given.
- "For the Nanopore 20X coverage, which is roughly equivalent to one ONT Flow cell of a human genome": this is for an ONT PromethION flow cell. A minION/GridION flowcells give only like 5X.
- A table similar to Table 1 for simulated data would be useful as a supplementary table.

- In Table 1, the accuracy values change substantially across different coverages. Will the accuracy values be observed consistently across different nanopore datasets for a given coverage?

Page 9

- "Vulcan by default uses a 90% threshold, yielding up to a 5% improvement in F1 score on low coverage (10X) ONT data.": As per Table 1 it is 3.79%, not 5%.
- "One difference between these two datasets is that the coverage of the PacBio CLR dataset is lower, and so the Sniffles minimum read support is set lower. Then when increasing the cut-off percentiles for Vulcan, there remain enough NGMLR mappings to meet or exceed the minimum read number support for SV calling.": This part was a bit unclear to me. Please consider increasing the clarity if possible/
- "Our results show that Vulcan runs up to 4X faster than NGMLR alone and produces lower edit distance alignments than minimap2, on both simulated and real datasets.": Only one set of benchmarks have been done (in Fig. 3 for Pacbio simulated). Where is the result for real data?

Page 11

- "multiprocessing module for multithreading support." : multiprocessing module for multicore support would be more technically accurate.
- As I understood primary mappings were kept and secondary mappings were ignored. What about the supplementary mappings?
- is user CPU time the accumulation of both user-time and system CPU time or just the user time?
- The terms speedup and runtime are not really compatible with CPU time. Instead, they relate more to wall clock time as mentioned earlier.
- "time command in Linux": Is this the inbuilt time command in Linux Bash or GNU time utility (available under /usr/bin/time)?
- "Furthermore, in order to profile the individual steps of Vulcan, we also counted the time usage per step on PacBio 30X coverage dataset with 90% percentile normalized edit distance cut-off.": Is it the 20% coverage dataset instead of 30% (as it is stated that "We chose PacBio CLR reads with 20X coverage as test data" at the beginning)?
- "Furthermore, in order to profile the individual steps of Vulcan, we also counted the time usage per step": How were these measured? "time" command cannot get this kind of granular details as far as I am aware.
- Which was the version of Truvari used?

Page 12

- What is the RAM availability on the computer used?

Supplementary information:

- Wasn't -MD used for minimap2?
- The log output from Vulcan did not have -x map-ont when I ran. See below:

Command:

vulcan -ont -t 32 -r ref.fa -i reads.fastq -w vulcan_tmp -o ./output-vulcan/vulcan

Log:

Executing: minimap2 -a --MD -t 32 -o minimap2_full.sam ref.fa / reads.fastq

Some typographical errors:

- Fig 1: some fonts are too small to read even on an A3 print

- throughout the paper the space preceding a citation is inconsistent: for example LAST [21] vs LRA[23] which is just a single example.
- page 1: (Sequel II -> bracket "(" seems to be not closed
- Fig 2: it reads like minimap2 Methods. Some vertical spacing between minimap2 and Methods is good.
- Page 11: Used by Valcan.. -> redundant.
- Page 11: asE = w/l. -> missing space.
- Page 11: Python3.8 -> space missing

Level of Interest

Please indicate how interesting you found the manuscript: Choose an item.

Quality of Written English

Please indicate the quality of language in the manuscript: Choose an item.

Declaration of Competing Interests

Please complete a declaration of competing interests, considering the following questions:

- Have you in the past five years received reimbursements, fees, funding, or salary from an organisation that may in any way gain or lose financially from the publication of this manuscript, either now or in the future?
- Do you hold any stocks or shares in an organisation that may in any way gain or lose financially from the publication of this manuscript, either now or in the future?
- Do you hold or are you currently applying for any patents relating to the content of the manuscript?
- Have you received reimbursements, fees, funding, or salary from an organization that holds or has applied for patents relating to the content of the manuscript?
- Do you have any other financial competing interests?
- Do you have any non-financial competing interests in relation to this paper?

If you can answer no to all of the above, write 'I declare that I have no competing interests' below. If your reply is yes to any, please give details below.

Potential conflicts of interesr: I have received travel and accommodation expenses to speak at Oxford Nanopore Technologies conferences.

I agree to the open peer review policy of the journal. I understand that my name will be included on my report to the authors and, if the manuscript is accepted for publication, my named report including any attachments I upload will be posted on the website along with the authors' responses. I agree for my

report to be made available under an Open Access Creative Commons CC-BY license (http://creativecommons.org/licenses/by/4.0/). I understand that any comments which I do not wish to be included in my named report can be included as confidential comments to the editors, which will not be published.

Choose an item.

To further support our reviewers, we have joined with Publons, where you can gain additional credit to further highlight your hard work (see: https://publons.com/journal/530/gigascience). On publication of this paper, your review will be automatically added to Publons, you can then choose whether or not to claim your Publons credit. I understand this statement.

Yes Choose an item.
