# Supplementary material for: Vulcan: Improved long-read mapping and structural variant calling via dual-mode alignment
Source: Gigascience. 2021 Sep 24;10(9):giab063. doi: 10.1093/gigascience/giab063 (PMC8463296; doi:10.1093/gigascience/giab063)
Supplement: giab063_Reviewer_2_Report_Original_Submission — Arang Rhie -- 7/10/2021 Reviewed [file giab063_reviewer_2_report_original_submission.pdf]

Reviewer Report

Title: Vulcan: Improved long-read mapping and structural variant calling via dual-mode alignment

Version: Original Submission **Date:** 7/10/2021

Reviewer name: Arang Rhie

Reviewer Comments to Author:

The authors present Vulcan, a combined alignment strategy that takes advantage of two mappers; Minimap2 and NGMLR. Minimap2 is known for its speed, while NGLR allows precise breakpoint detection from more accurate alignments. The introduction and conducted experiment are sound and results look promising; however, addressing a few points laid below will improve the manuscript and help readers to understand.

1. A clear definition of the edit distance cutoff in the results with more details in the Methods would help understanding without jumping around the manuscript. Currently, on page 4, the text states "by thresholding the edit distance from the mappers" and redirects to the Methods, requiring readers to jump over to Methods. The cutoff is mentioned as i.e. "90% cutoff", which is confusing if not seen the Methods first - does this mean a read with >90% of the bases being 'edit's? or is this <90%?
2. The edit distance (NM tag) in minimap2 does not include the number of soft/hard clipped bases. A lot of SV breakpoints cause read clippings in Minimap2; which could be rescued with NGMLR's convex scoring scheme. I'd be curious if this will improve total recall and precision if considered.
3. The result section seems unorganized with too many sub-headings
4. Cyp2d6 is mentioned as a gene; capitalize and italicize all gene names accordingly. i.e., CYP2D6 in italic
5. Provide the ONT, HiFi, CLR coverage used for HG002 in the Data Description
6. A few sentences are incomplete or grammatically incorrect. I'd recommend the authors to go through and take another careful look for those. i.e. page 3, "The key idea behind Vulcan is to identify reads that are sub-optimally aligned based on edit distance and then realign them with a more sensitive ?? (NGMLR by default)."
7. Inconsistent coverage abbreviation: x and X are used inconsistently. i.e. 20x, 30x, 50X on page 7.
8. Genome in a bottle is first mentioned on page 3 as "GIAB". Spell it out on page 3 and use the abbreviation on page 6. Similarly, SV is first well declared, however mentioned as "Structural Variants" in a lot of places along with the abbreviated "SV". It is also inconsistently used among the sub-heading.

Level of Interest

Please indicate how interesting you found the manuscript: Choose an item.

Quality of Written English

Please indicate the quality of language in the manuscript: Choose an item.

Declaration of Competing Interests

Please complete a declaration of competing interests, considering the following questions:

- Have you in the past five years received reimbursements, fees, funding, or salary from an organisation that may in any way gain or lose financially from the publication of this manuscript, either now or in the future?
- Do you hold any stocks or shares in an organisation that may in any way gain or lose financially from the publication of this manuscript, either now or in the future?
- Do you hold or are you currently applying for any patents relating to the content of the manuscript?
- Have you received reimbursements, fees, funding, or salary from an organization that holds or has applied for patents relating to the content of the manuscript?
- Do you have any other financial competing interests?
- Do you have any non-financial competing interests in relation to this paper?

If you can answer no to all of the above, write 'I declare that I have no competing interests' below. If your reply is yes to any, please give details below.

I declare that I have no competing interests

I agree to the open peer review policy of the journal. I understand that my name will be included on my report to the authors and, if the manuscript is accepted for publication, my named report including any attachments I upload will be posted on the website along with the authors' responses. I agree for my report to be made available under an Open Access Creative Commons CC-BY license (http://creativecommons.org/licenses/by/4.0/). I understand that any comments which I do not wish to be included in my named report can be included as confidential comments to the editors, which will not be published.

Choose an item.

To further support our reviewers, we have joined with Publons, where you can gain additional credit to further highlight your hard work (see: https://publons.com/journal/530/gigascience). On publication of this paper, your review will be automatically added to Publons, you can then choose whether or not to claim your Publons credit. I understand this statement.

Yes Choose an item.
